# Multivariate Analytical Approaches to Identify Key Molecular Properties of Vehicles, Permeants and Membranes That Affect Permeation through Membranes

**DOI:** 10.3390/pharmaceutics12100958

**Published:** 2020-10-11

**Authors:** Omaima N. Najib, Stewart B. Kirton, Gary P. Martin, Michelle J. Botha, Al-Sayed Sallam, Darragh Murnane

**Affiliations:** 1 Institute of Pharmaceutical Science, Franklin Wilkin’s Building, King’s College London, 150 Stamford Street, London SE1 9NH, UK; omaima_najib@hotmail.com (O.N.N.); gary.martin@kcl.ac.uk (G.P.M.); 2 International Pharmaceutical Research Centre, 1 Queen Rania Street, Amman 11196, Jordan; 3 Department of Clinical, Pharmaceutical Science and Biological Sciences, University of Hertfordshire, College Lane, Hatfield AL10 9AB, UK; s.b.kirton3@herts.ac.uk (S.B.K.); m.botha@herts.ac.uk (M.J.B.); 4 Al-Taqaddom Pharmaceutical Industries, Co. 29-Queen Alia Street, Amman 11947, Jordan; a.sallam@tqpharma.com

**Keywords:** PCA, QSPR, permeability, membrane interaction, oily vehicle, enhancer of drug penetration

## Abstract

There has been considerable recent interest in employing computer models to investigate the relationship between the structure of a molecule and its dermal penetration. Molecular permeation across the epidermis has previously been demonstrated to be determined by a number of physicochemical properties, for example, the lipophilicity, molecular weight and hydrogen bonding ability of the permeant. However little attention has been paid to modeling the combined effects of permeant properties in tandem with the properties of vehicles used to deliver those permeants or to whether data obtained using synthetic membranes can be correlated with those obtained using human epidermis. This work uses Principal Components Analysis (PCA) to demonstrate that, for studies of the diffusion of three model permeants (caffeine, methyl paraben and butyl paraben) through synthetic membranes, it is the properties of the oily vehicle in which they are applied that dominated the rates of permeation and flux. Simple robust and predictive descriptor-based quantitative structure–permeability relationship (QSPR) models have been developed to support these findings by utilizing physicochemical descriptors of the oily vehicles to quantify the differences in flux and permeation of the model compounds. Interestingly, PCA showed that, for the flux of co-applied model permeants through human epidermis, the permeation of the model permeants was better described by a balance between the physicochemical properties of the vehicle and the permeant rather than being dominated solely by the vehicle properties as in the case of synthetic model membranes. The important influence of permeant solubility in the vehicle along with the solvent uptake on overall permeant diffusion into the membrane was substantiated. These results confirm that care must be taken in interpreting permeation data when synthetic membranes are employed as surrogates for human epidermis; they also demonstrate the importance of considering not only the permeant properties but also those of both vehicle and membrane when arriving at any conclusions relating to permeation data.

## 1. Introduction

The in vitro and in vivo experimental measurement of skin permeability is a difficult and complex process, due in part to ethical concerns regarding human and animal experimentation. As a consequence, there has been a great interest in recent years to avoid unnecessary, time consuming and costly in vitro testing by developing models that would allow formulation scientists to predict the ability of a compound to cross the skin based on its physicochemical characteristics.

One approach used to identify this relationship between molecular properties and skin permeation is multivariate analysis. A number of attempts have been made to develop quantitative structure–permeability relationship (QSPR) models of skin permeability by seeking linear correlations between observed experimental values for permeation and the physicochemical properties and/or molecular structure parameters of the chemical compounds being studied (see, e.g., [1,2,3,4,5,6,7,8,9]). Such models have been used by environmental agencies for the safety assessment of dermal exposure to industrial and environmental hazards, and also by pharmaceutical companies to screen and select drugs for possible transdermal delivery potential. 

Permeability through the skin is thought to be controlled by the physicochemical properties of both the permeant and the vehicles used to deliver it. For example, the chemical structure of a vehicle can determine the effect that it is likely to have on membranes and/or on the partitioning of the penetrant, leading to the observed changes in the skin penetration profile. However, most previous predictive models of permeability have largely concentrated on the chemical structure of the permeant and have not considered the effect of either vehicle or membrane on the diffusion process (see e.g., [2,10]). Many of the developed QSPR models are based on a database assembled by Flynn [1]. It is comprised from 97 human skin permeability coefficients for 94 compounds obtained in vitro through human skin from 15 different literature sources. Nevertheless, previous experiments have demonstrated that the vehicle and the membrane are as important as the permeant, since they are able to enhance or retard the permeation by virtue of their physical and chemical properties [11]. Different studies have showed that vehicles penetrate into the membranes and skin, affecting it through different mechanisms; these involve disruption of the intercellular lipids of the highly ordered stratum corneum (SC), interaction with intracellular proteins, or improvement of the drug partition into the stratum SC, including lipid fluidization, disruption of lipid structure, lipid extraction and also irreversible protein denaturation in SC [12,13].

Applications of Principal Components Analysis (PCA) have found widespread use in most stages of pharmaceutical research and industry. For example, it has been used in distinguishing, quantifying and predicting the compression behavior of pure excipients and binary blends on tablet formation and properties [14], and formulation scientists have used such analysis for characterizing the performance of nebulizers [15]. In addition, PCA has been used to study the relative importance of certain structural characteristics in terms of their contribution to transdermal permeation [9]; and classification of drugs according to their mode of action [16]. The application of multivariate analysis to experimental data obtained for a number of different penetrants, in a range of vehicles across synthetic membranes and epidermis, could provide an important insight into possible means of enhancing dermal delivery, leading to the development of new therapeutic formulations. Therefore, there is an interest in the construction of prediction models for skin permeability since most existing models are based on linear algorithms, although nonlinear models applying nonlinear regression techniques, including artificial neural networks or support vector regression and random forest, have also been developed and applied to QSPR models [17,18,19,20]. Several authors have worked on different mathematical computer-generated penetration models to predict the release, flux, or skin permeability of different topical formulations [21,22,23]. However, for the development of robust and predictive QSPR models, it is important that all experimental data be consistent and reliable. Ideally, all experiments should be performed under standardized conditions, such as employing the same skin type from the same anatomical region and maintaining constant laboratory conditions such as temperature, permeant concentration and receptor medium. Practically, this has not been the case; therefore, a potential limitation of existing QSPR models of skin permeation is that the data used to build and validate them are derived from an extensive database from different investigators and laboratories using disparate experimental protocols, which could undermine the generalizability of a model.

Hence, the aim of this study was to use multivariate analyses on a self-consistent set of experiments to better understand the influence of physicochemical properties of permeants, oily vehicles and membranes on: solvent uptake by membranes (i.e., a vehicle–membrane interaction) and the flux and permeability coefficient of the compounds moving through those membranes.

## 2. Materials and Methods

### 2.1. Materials

Methanol, acetonitrile and hexadecane (HD) were obtained from Merck chemicals, Darmstadt, Germany; orthophosphoric acid from Riedel-de-Haen Chemical, Seelze, Germany; potassium hydroxide from Scharlau Chemical, Barcelona, Spain; methyl paraben (MP), butyl paraben (BP), caffeine (CF) and triethylamine from Sigma Chemical Co., Gillingham, UK; oleic acid (OA) and liquid paraffin (LP) from Sigma, Taufkirchen, Germany; isopropanol from BDH Laboratory Supplies, Poole, UK; isopropylmyristate (IPM) and isohexadecane (IHD) from Uniqema, Klang, Malaysia; and silicone membrane from Samco Ltd., Nuneaton, UK. Whatman no. 1 filter paper was purchased from Whatman International Ltd, Maidstone, UK. High-density polyethylene (HDPE) membrane (pharmaceutical grade package material) and polyurethane (PU) membrane were donated by TQ pharma, Amman, Jordan and Exopack, Wrexham, UK, respectively.

### 2.2. HPLC Chromatographic Conditions for Model Permeants

A Class VP 2010 LC Pump with Auto sampler connected to a UV Absorbance Detector were employed (Shimadzu, Kyoto, Japan). The column used for all permeants was a Symmetry 5 μm BDS (C18), 150 × 4.6 mm (5 μm) (Waters, Milford, MA., USA). The mobile phase for MP consisted of 35% acetonitrile/65% *v*/*v* phosphate buffer (50 mM KH_2_PO_4_ containing 1% *w*/*v* triethylamine, then adjusted to pH 3.5 with orthophosphoric acid). The mobile phase for the assay of BP was 50% acetonitrile/50% phosphate buffer (50 mM KH_2_PO_4_ adjusted to pH 3.0 with orthophosphoric acid) and for CF was 15% acetonitrile/85% *v*/*v* phosphate buffer (50 mM KH_2_PO_4_ adjusted to pH 3.0 with orthophosphoric acid). The flow rate was maintained at 1 mL min^−1^ and injection volumes were 10 µL for MP and BP and 50 µL for CF. The wavelength of detection (nm) was 254, 256 and 270 for MP, BP and CF respectively (*n* ≥ 4).

### 2.3. Franz Cells Studies-Synthetic Membranes.

The protocol for the Franz cell experiments using synthetic membranes are described in detail elsewhere [24]. 

### 2.4. Franz Cell Studies Human Epidermis

Fresh, surgically excised samples of human skin were obtained directly after abdomino-plastic surgery carried out on a 35-year-old Middle Eastern female patient attending a private clinic in Amman, Jordan, with informed consent. The board of directors and the ethics committee of Dawoud Clinic (Amman 11182, Jordan) reviewed and approved the informed consent (TDRE-001 approved on 01/02/2011). Subcutaneous fat was carefully removed from the skin sample using forceps and a scalpel. Following the removal of the fat, individual portions of skin were immersed in water at 60 °C for 45 s. The skin was then pinned, dermis side down, on a corkboard, and the epidermis (comprising stratum corneum and epidermal layer) was gently removed by rolling the membrane using forceps. The dermis was then discarded, and the epidermal membrane floated onto the surface of water and was taken up onto a Whatman no.1 filter paper. The resultant epidermal sheets were blotted dry with tissue paper, wrapped in aluminium foil and stored flat at −70 °C until use, and checked for integrity before use. 

In order to determine the extent to which permeant bound to the filter paper, circular pieces of filter paper were immersed in 5 mL of a standard solution (10 µg mL^−1^) of each permeant in phosphate buffer. Samples (200 µL) were removed from each vial after 0.5, 4, 6 and 8 h and assayed using HPLC.

The permeation of MP, BP and CF from either the oily vehicles or orthophosphate buffer (pH 7.0) was determined as follows. The receptor compartment of a Franz cell was carefully filled with phosphate buffer, the temperature of which was maintained at 32 °C. A small Teflon-coated magnetic bar (around 5 mm) was included in the receptor compartment such that stirring was maintained throughout the duration of the experiment. String rate and temperature were fixed during the entire course of the experiment. The epidermal membrane was then allowed to equilibrate with the receptor fluid for 1 h, after which 200 μL of the oily suspension was introduced onto the stratum corneum (i.e., donor compartment). The duration of each skin permeation experiment was 8 h for MP and BP and 10 h for CF. The permeant concentration of receptor samples was analysed by HPLC (*n* ≥ 4).

The concentration of the permeant in the receptor solution at any time point was corrected for previous sample removal. The cumulative amount of permeant crossing the epidermis per unit of skin surface area was plotted as a function of time, and the slope of the linear portion of the plot was determined and taken as a measure of the average flux (J, µg cm^−2^ h^−1^). 

### 2.5. Data Pre-Treatment and Statistical Analyses 

Permeation data reported in this study are mean values (*n* ≥ 4) with accompanying standard deviation (sd). In order to establish differences in the parameters measured, statistical analysis of the data was undertaken using either analysis of variance (ANOVA) or Student’s *t*-test, as appropriate. Post hoc comparisons of the means of individual groups after carrying out ANOVA were performed using Tukey’s test. ANOVA and Post hoc Tukey’s test were performed using SYSTAT version 5.0 (SYSTAT Software Inc., San Jose, CA, USA), and the level of significance for results was taken at *p* ≤ 0.05 in all cases. 

### 2.6. Principal Component Analysis 

Molecular descriptors for the penetrants, vehicles and membranes used in the PCA were calculated using Molecular Operating Environment 2012, (Chemical Computing Group Inc., Montreal, QC, Canada), and Hansen solubility parameters (HSP) by the HSPiP software (version 4.0.0.4, www.hansen-solubility.com). All abbreviations are defined in Appendix I. These included δ_D_, δ_P_, δ_H_, solubility parameter MPa^½^, HSP distance (Equation (1)) between the oils and membranes; Mvol, Mwt, density, Mpoint, Log K_o/w_, ovality, molecular connectivity index, weight, Log P, TPSA, vdw_vol, vdw_area, a_nH, a_nC, a_nO, b_1rotN, b_1rotR, b_count, b_double, b_rotN, b_rotR, b_single, lip_acc, lip_don, opr_brigid, opr_nrot, chi0, chi0_C, chi1, chi1_C, chi0v, chi0v_C, Kier1, Kier2, Kier3, Kier flex, balabanJ, Q_VSA_HYD_PEOE_VSA_HYD, b_ar, opr_nring, Q_VSA_POL_PEOE_VSA_POL, Q_VSA_FHYD_PEOE_VSA_FHYD, Q_VSA_FPOL_PEOE_ and VSA_FPOL. The HSP distance between the oils and membranes was calculated using Equation (1):

(1) $$Distance^{2}=4{\left(\delta \mathrm{DA}-\delta \mathrm{DB}\right)}^{2}+{\left(\delta \mathrm{PA}-\delta \mathrm{PB}\right)}^{2}+{\left(\delta \mathrm{HA}-\delta \mathrm{HB}\right)}^{2},$$

where A and B are oils and membranes, respectively.

PCA of the descriptors was carried out using Unscrambler X (CAMO software, Oslo, Norway) for four experiments: solvent uptake, flux and K_p_, for synthetic membranes and flux alone through epidermis. Identification of important descriptors for individual experiments was achieved in an iterative manner as follows. PCA loadings plots for each of the experiments were analyzed and the descriptor that had the lowest absolute value on PC-1 in the plot was removed. The rationale for this is that such a descriptor does not contribute to the explanation of the experimental variance in the PCA model. The PCA model was then regenerated using the remaining input variables. This process was repeated until only the descriptors relevant to explaining the variance in the dataset remained. If, upon removal of a variable, there was a decrease in the percentage of explained variance seen in PC-1, or a loss of orthogonality between the principal components (PCs), then the descriptor was returned to the model and the process terminated, as the minimal number of variables required to explain the variance in the dataset had been identified.

### 2.7. Generation and Evaluation of QSPR Models

**Figure 1:**
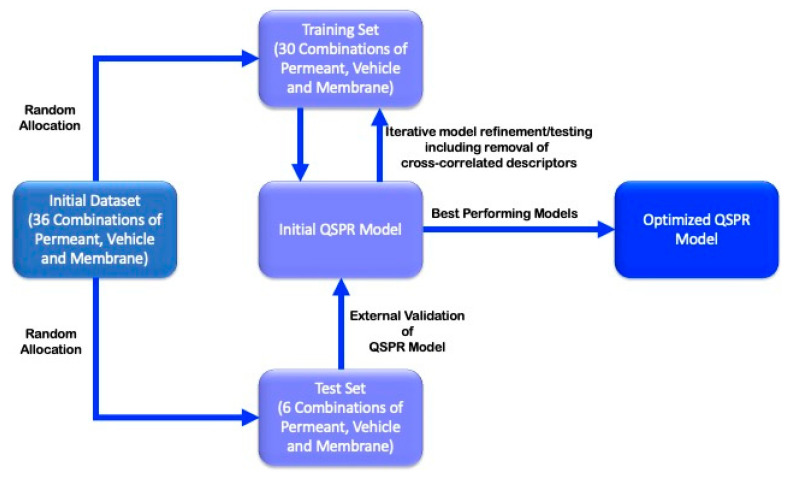
Graphical overview of the quantitative structure–permeability relationship (QSPR) modelling process.

QSPR models to predict flux and permeation coefficients across synthetic membranes were generated and evaluated using the QuaSar module in the molecular operating environment (MOE) software. To this end, the initial dataset (36 observations, corresponding to 36 combinations of permeant/vehicle/membrane) was divided into training (30 observations) and test sets (6 observations), ensuring that the test set was representative of the range of membranes, oily vehicles and permeants investigated. The data analytical workflow is presented graphically in Figure 1.

A cross-correlation matrix for all of the descriptors given in Section 2.6 was generated. If two descriptors demonstrated absolute correlation coefficient values above 70% with one another, the descriptor least correlated with the dependent variable (either flux or permeation coefficient) was removed from the analysis. This was carried out to guard against the over-representation of related molecular properties in the final models. The remaining descriptors were then subjected to zero mean unit variance scaling. 

QSPR models were constructed using the estimate linear model (ELM) equation and the Estimated Normalized Linear Model (ENLM) algorithms in the QuaSAR module in MOE. A correlation coefficient (r^2^ value) and the cross-validated correlation coefficient (q^2^ value) were generated and used as measures of model quality and robustness, respectively. Iterative refinement of initial models was achieved by removing the molecular descriptor in the equation identified as contributing least to the initial model and regenerating the QSPR equation. This process was carried out until the r^2^ and q^2^ values were in close proximity, and the model possessed the highest r^2^ values possible with the least number of molecular descriptors. The r^2^ values obtained from the test set was then used to assess the predictive quality of the refined models (Figure 1).

## 3. Results

### 3.1. Effect of Vehicle on Diffusion through Human Epidermis

The amount of drug binding to the filter paper on which the epidermis was supported during the Franz cell study was found to be negligible for all three penetrants (data not shown). The parameters derived from the in vitro epidermal diffusion studies are shown in Table 1. The fluxes and permeability coefficients of model permeants penetrating the skin when the compounds were applied in IHD as a vehicle were found to be significantly higher (*p* ≤ 0.05) than those which diffused from any other vehicles. IHD is small in size and has high flexibility compared to other oils. The mean flux values were in the order of MP > BP > CF. Nevertheless, all oils significantly enhanced the diffusion of model permeants through the human epidermis compared with phosphate buffer (ANOVA and Tukey’s test; *p* < 0.05).

### 3.2. Principal Component Analysis

Hotelling’s test [25,26] showed that there were no outliers in the dataset. The data obtained using liquid paraffin (LP) as a vehicle were not included in these analyses, as this vehicle is comprised of a mixture of disparate molecular components (of poorly defined composition) and is therefore difficult to accurately characterize using the available molecular descriptors [27]. Molecular descriptors for the penetrants, vehicles and membrane is essential to explaining the variation in the measurements in each of the four experiments, as determined by PCA, are summarized in Table 2.

### 3.3. PCA Analyses: Synthetic Membranes

#### 3.3.1. Solvent Uptake

**Figure 2:**
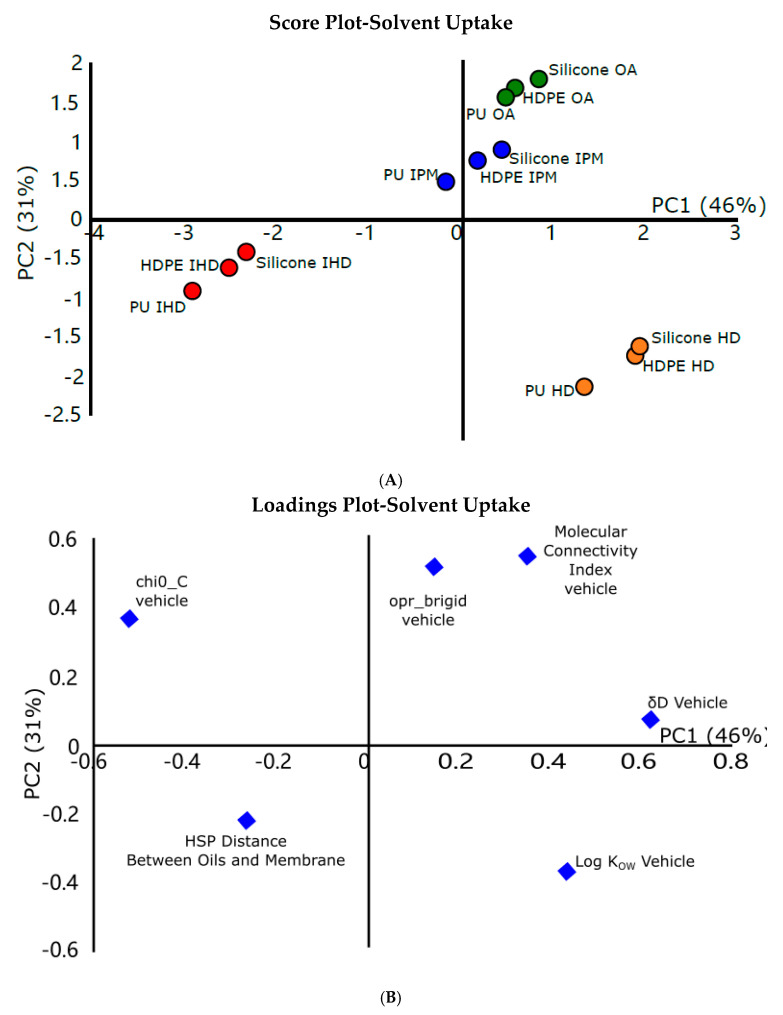
(**A**) Scores plot from the PCA of solvent uptake of oily vehicles into synthetic membranes showing clear groupings with respect to different vehicles (isohexadecane (IHD) in red, hexadecane (HD) in orange, oleic acid (OA) in green and isopropylmyristate (IPM) in blue). The first two principal components (PCs) explain 77% of the total variance in the dataset; 46% and 31% of the variability are explained by PC1 and PC2, respectively. (**B**) Loading plots from the PCA of the solvent uptake of oily vehicles into synthetic membranes showing descriptors, primarily related to describing the physicochemical properties of the vehicle, being highly loaded on PCs 1 and 2 and hence being most responsible for explaining the variation in solvent uptake observed.

An examination of the model for solvent uptake shows that the first two PCs in this experiment describe 77% of the total variance in the data (Figure 2). The molecular descriptors δ_Dm_ and δ_Tm_ were removed from the analysis because the initial results showed them to be so different to the other descriptors that they skewed the analysis. This was not surprising, since only a limited number of membranes (three) with vastly different solubility parameters were under study. However, despite δ_Dm_ and δ_Tm_ not loading heavily on either PC1 or PC2, these were the only parameters in the initial descriptor set that represented the properties of the membrane. If this study is to be robust, membrane descriptors should not be excluded, and so an alternative was needed. To this end, the distance between the Hansen Solubility Parameter (HSP) values of the membranes and vehicles, was introduced. This derived parameter represents a measure of the degree of matching (i.e., similarity) between the solubility parameters of the vehicles and the membranes [28]. The lower the HSP distance value, the more alike the polymer (membrane) and solvent (oily vehicle). Therefore, the vehicle that has similar partial components to the partial components of the membrane might be expected to induce the greatest swelling of the membrane, consequently modifying the partitioning and diffusion of permeants through the membrane. Therefore, HSP distance represents a descriptor relating the vehicle to the membrane (i.e., a vehicle–membrane interaction term). HSP distance was shown by PCA to be significant in its contribution to explaining the variance of the data with respect to solvent uptake. 

The scores plot (Figure 2A) shows that there was no significant clustering of data with respect to the membrane used, i.e., each membrane investigated was found in all of the different groups on the plot. In contrast, the clustering was clearly correlated with the oily vehicle used. Hence, these findings suggest that variation in the extent of solvent uptake in synthetic membranes is dominated by the properties of the vehicle used to deliver the penetrant. This is supported by analysis of the associated loadings plot (Figure 2B), which shows that the majority of the descriptors that have high loading values on PC1 and PC2 are describing the properties of the oily vehicle.

The scores plot (Figure 2A) shows the four oily vehicles cluster into three distinct groups. IPM and OA, both of which contain heteroatoms which render them more polar than IHD and HD, are closely grouped together in the upper right-hand quadrant of the plot. IHD and HD, which are simple hydrocarbons, occupied their own distinct regions in the scores plot. The separation of HD and IHD into two groups may appear initially surprising, given that HD is the linear counterpart of IHD. In fact, the separation into two groups implies that the combination of descriptors which make up PC1 and PC2 are identifying differences in the vehicles based on both electronic and steric properties. This is supported by an examination of the loadings plots which, for example, show that the high loading of chi0_C, (a topological descriptor encoding the size, shape and flexibility), on both PC1 and PC2 is important for explaining the variance observed in the experimental results. This suggests that there is a correlation between the size, flexibility and the ability to disrupt and swell the membrane, which was also confirmed by the flux results of permeants from different oils through the membranes. 

#### 3.3.2. PCA Analyses of Flux and Permeation Coefficient (K_P_) in Synthetic Membranes 

**Figure 3:**
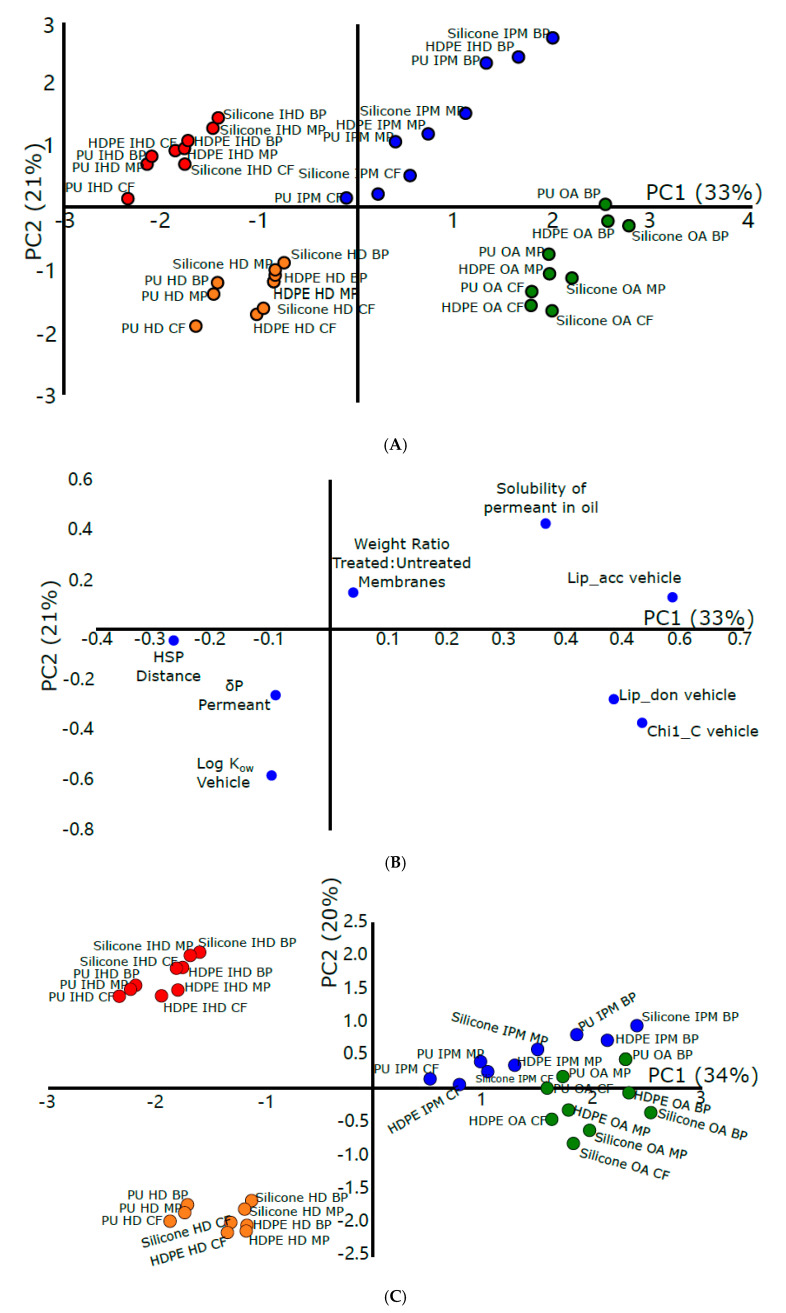

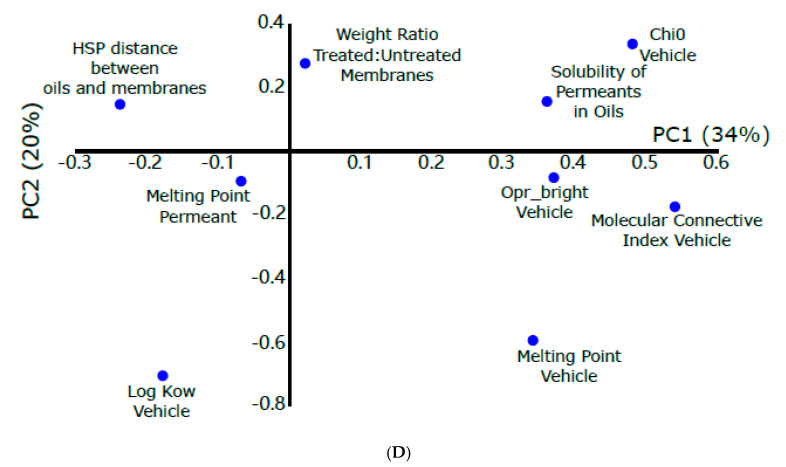
(**A**) Scores plot from the PCA of flux and (**C**) scores plot from the PCA of permeation coefficient through synthetic membranes showing clear groupings with respect to the different vehicles used (IHD in red, HD in orange, OA in green and IPM in blue). (**B**) Loading plots from the PCA of flux and (**D**) loading plots from the PCA of permeation coefficient across synthetic membranes showing high loadings for descriptors primarily related to the physicochemical properties of the vehicle and the solubility of the permeants in the vehicle contributing most to explaining the variance observed in the datasets.

PCA experiments incorporating the molecular descriptors identified as being important for explaining the variance in flux and permeation across synthetic membranes (Table 2) were carried out. The data in the scores plot for flux (Figure 3A) are distributed into four groups, again clustered according to the vehicle used, with the identity of the membrane and permeant seemingly less important for explaining the variance in the dataset. The loading plot for flux (Figure 3B) shows that the H-bond acceptor of the vehicle has the greatest impact of the included descriptors in describing the variance of the dataset. Chi1_C, a steric descriptor relating to localized branching within a molecule, is also highly loaded indicating the importance of the overall topology of the oil in describing variation in the dataset. The log K_o/w_ vehicle and solubility of the permeants in the oils are the descriptors which are most highly loaded on PC2. These distributions of variables on both PCs indicate that the flexibility and ease of interaction between the oil and membrane has a key role in the permeation through membrane in addition to the solubility of the permeant in the oil. This suggests that the flux is driven by the match between the oil where the drug should be soluble in the modified membrane. On the other hand, if the drug solubility is low, the solvent might permeate into the skin leaving the drug on the surface. The K_p_ score plot (Figure 3C) shows that the data are clustered into three groups. One group consists of the experiments carried out using IPM and OA, the second contains the HD data and the third the IHD data. In the K_p_ loading plot (Figure 3D), the molecular connectivity index for the vehicle, a steric descriptor, was shown to have had the biggest effect in explaining the variance of the data captured by PC1. Chi0 (vehicle), another steric descriptor, also contributes significantly to explaining the variance captured by PC1. This descriptor is mainly related to the shape and bonding of the vehicle indicating the importance of vehicle shape and flexibility in the permeation and disruption of the membrane. PC2 showed high loadings for Log K_o/w_ (vehicle), a lipophilic descriptor. IPM and OA have similar values for their molecular connectivity indices and Chi0 descriptors, and so their proximity on the scores plot in this PCA is not surprising. 

Hence, the parameters describing variance in flux and Kp in the dataset are lipophilic and steric in nature. Once again, the loading plots are dominated by descriptors relating to the properties of the oily vehicle. This indicates the impact of the vehicle in the flux process and is evidence to support the initial concerns regarding the limitations of existing models of flux that only take the identity of the permeant into account. Therefore, it can be assumed that the flexibility and size of the vehicle will aid in its permeation through the membrane provided that there is a match of lipophilicity between the membrane and vehicle. This was confirmed by the results of membrane swelling data with IHD, which has the smallest and most flexible structure compared to other vehicles, since it was highly sorbed to/into the membrane and enhanced the flux of model permeants. In contrast, oleic acid, which has the largest molecular structure and is the least flexible, led to less swelling of the membrane and less permeation of the permeants.

#### 3.3.3. PCA Analysis of Flux through Epidermis 

**Figure 4:**
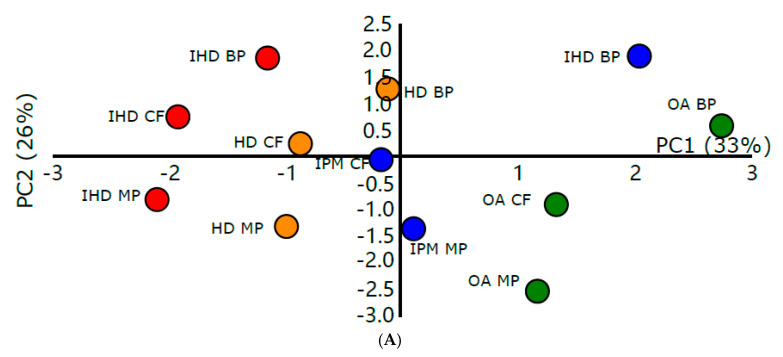

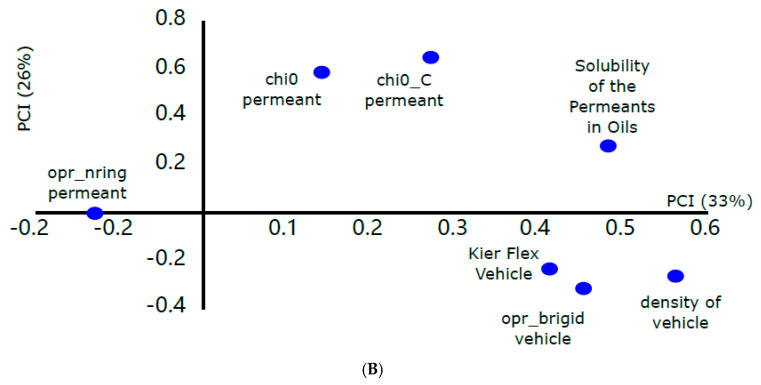
(**A**) Score plot from PCA showing the relationship between permeant and oily vehicle descriptors with epidermis flux. The two displayed PCs explain 33% and 26% of the variance, respectively. (**B**) Loading plots from PCA showing the relationship between permeant-oily vehicle descriptors with epidermis flux.

A PCA was carried out on the permeant and vehicle molecular descriptors for the flux of the model compounds through human epidermis (Table 2, Figure 4). The first two PCs describe 59% of the variance in the data. The scores plot shows that, unlike the synthetic membrane flux data, there is no tight clustering according the identity of the vehicle. There is a pattern of separation that appears to be due to both the identity of the permeant and the vehicle, although the flux of BP from IPM skews this distribution. This phenomenon might be a consequence of the comparatively high solubility of BP in IPM (120.8 ± 2.6 mg mL^−1^) relative to the solubility of BP in the other oily vehicles.

Upon examination of the loadings plot, the density of the vehicle, the solubility of the permeant in the oil and opr-brigid, a steric descriptor capturing the relative rigidity of the vehicle resistance to rotate along the vehicle molecular bond which will affect the flexibility of the movement, were found to have the greatest loadings on PC1 with Chi0_C and chi0 values of the permeant being heavily loaded on PC2. In contrast to the studies on synthetic membranes, this showed the importance of both the identity of the permeant and the identity of the vehicle in determining flux in the epidermis studies. The flux of the model permeants (MP > BP > CF) from the buffer across human epidermis reflected the differences in lipophilicity of each of the permeants and the ease of the compounds in traversing the skin barrier. 

All oily vehicles enhanced the permeation of the model permeants from the saturated solutions through the human epidermis relative to the buffer. The factors affecting the diffusion of permeants from these vehicles may include differences in either (i) vehicle–skin effects and/or (ii) permeant–vehicle affinity. Using a cellulose acetate nitrate membrane (non-rate limiting), the highest permeation was attained using IHD. IHD is highly branched molecule which is more flexible than other vehicles (Kier, 1989). Such results support the information provided by the loadings plot that the shape and size of the vehicle is a key determinant in permeation of the small molecule. 

The distribution of variables on both PCs indicate that for flux to be achieved the oils should be small and flexible so as to gain access to the membrane, disrupt the packing and modify it. The solubility of the permeant in the solvent and the uptake of the solvent into the membrane were found to have an influence on the flux. The permeant should have a high solubility in the oil that has entered the membrane for the permeant to partition into the membrane. The permeant also should have high solubility and diffusivity in the vehicle–membrane phase to drive permeation across the membrane. The results of the solvent uptake and diffusion studies indicated that solvents which were taken up to a significant extent into the membrane caused the membrane to swell and led to higher diffusion of the permeant through the membrane.

### 3.4. Quantitative Structure-Property Relationship Models: 

The initial three-descriptor ENLM QSPR (Estimated Normalized Linear Model Quantitative Structure Property Relationship) model generated to account for the permeation coefficient (*K_p_*) through synthetic membranes had the form:

(2) $$K_{p}=0.41902\;solubility-0.85524\;lip\_acc\;vehicle+0.64106\;\delta H_{exp\;drug}+0.6976,$$

where *solubility* represents the *solubility* of the permeant in the oily vehicle, *lip_acc vehicle* is the number acceptors, as defined by Lipinski’s rules, for the oily vehicle and *δH_exp drug_* is a Hansen solubility parameter that describes the energy due to hydrogen bonding of the permeant molecules.

The three-descriptor model gave an r^2^ value of 0.67 and a q^2^ value of 0.59 for the training set and produced an r^2^ value of 0.56 for the test set. These values suggest that the model is relatively predictive, and reasonably robust given the small differences between r^2^ and q^2^ values. This is encouraging given the relatively small dataset. In addition, the fact that the training set q^2^ value is lower than the r^2^ value, and the r^2^ value of the test set is lower than that for the training set indicates the model is not overfitted, i.e., it is reasonable to assume that it is a generally applicable model.

The ENLM QSPR model generated to predict normalized flux (i.e., flux corrected for the thickness of the synthetic membrane) gave the following six-descriptor equation:

Flux = 1.08083 + 0.23534 × weight ratio − 0.36141 × log K_o/w_ vehicle − 0.24036 × MPt_permeant_ − 1.01839 × δH_exp membrane_ + 0.47908 × δP_exp membrane_ − 0.34680 × normalized opr_brigid_vehicle_ (3)

where δH_exp membrane_ and δP_exp membrane_ are Hansen solubility parameters related to the energy due to hydrogen bonding and dipolar interactions in the membrane, respectively, log K_o/w vehicle_ relates to the octanol-water partition coefficient of the oily vehicle, weight ratio refers to the weight of the swollen synthetic membrane in comparison to the original membrane weight, MPt_permeant_ is the melting point of the permeant and opr_brigid_vehicle_ describes the number of rigid bonds in the oily vehicle, as defined by Oprea. This model reported a training set r^2^ of 0.72 and q^2^ of 0.59. The test set r^2^ value was 0.65, again indicating a relatively robust, predictive and generalizable model.

No attempt was made to build QSPR models for predicting flux through epidermis because of the small dataset (*n* = 9).

## 4. Discussion

### 4.1. Principal Component Analyses

The PCA results clearly demonstrate that the choice of vehicle (and hence the physicochemical properties of the vehicle) is the most important factor in accounting for the differences observed in permeation coefficients and flux across synthetic membranes. This was apparent from the clusters that form within the scores plot which are grouped according to the oily vehicles used in the experiment and the fact that the loadings plots show that the descriptors that are most heavily loaded, i.e., those that are most important in explaining the observed variance in the dataset, are related to the properties of the vehicle, such as size and flexibility, along with the physicochemical matching between the vehicle and the membrane.

This dominance of properties of the oily vehicle was not evident when examining the experiments which determined fluxes through human epidermis. In this instance, the experiments carried out indicate that the properties of both the permeant and the oily vehicle play a part in determining the rate of penetration of the compound. This stark difference between the behavior of synthetic membranes in relation to oily vehicles and the behavior of excised skin in relation to the same substances is of interest as it confirms the limitations of employing synthetic membranes as a surrogate for human excised skin models. It has been previously concluded that membranes fail to provide an analogous model for the complexity of the stratum corneum [29]. Nevertheless, several studies on different membranes have been conducted trying to mimic the behavior of the skin. Carbosil membrane which is composed of polydimethylsiloxane–polycarbonate block copolymer was shown to provide mechanistically based models of a good predictive capacity for percutaneous drug transport [30,31]. Like human skin epidermis, it has a heterophase and heteropolar structure; therefore, it was able to mimic the skin permeation barrier where the drug transport process is described by a solubility (partition)-diffusion process. The barrier function of the skin and carbosil was examined for a wide range of structures and therapeutic classes of the drugs where their permeability coefficients had a clear dependence of permeant properties alone (i.e., molecular weight, melting point, solubility in aqueous solution and octanol–water partition coefficient) [30,32].

Drug permeation through epidermis has been identified previously as possibly being dependent upon both the physicochemical properties of the drug and those of the vehicle comprising the formulation. In the case of the epidermis, the vehicle can interact with the membrane to change the phase behavior leading to fluidization of the intercellular lipids within the stratum corneum [12]. Other vehicles induce destabilization of the stratum corneum structure, and hence render it more susceptible to penetration by concomitantly applied permeants [33]. The use of vehicles that are sorbed by the membrane or interact with components of the membrane might induce changes in the physicochemical properties of the synthetic barriers due to the solvation of the polymer chains. Silicone membrane is cross-linked in structure, and it is likely that the weight gain observed following equilibration with specific oil is more associated with chain solvation and subsequent membrane swelling interactions rather than polymer dissolution which can occur with non-cross-linked barriers. Additionally, penetration-enhancing chemical agents are often co-formulated in vehicles, which serve to improve the permeability of the epidermal barrier to xenobiotics. The understanding of vehicle–membrane interactions is therefore a critical step not only in the selection of an optimal penetration enhancer but also a prime factor affecting formulation design. Investigation of a possible membrane–vehicle interaction, using solvent uptake, and then seeking to correlate this with the effect on drug permeation, has been employed by a number of previous workers, although almost exclusively employing aqueous- and alcoholic-based vehicles [34]. In the current study, it has been demonstrated that PCA provides an effective analytical tool to examine multiple interacting vehicle, drug and barrier descriptors to predict the influence of formulation on permeant flux across barrier membranes. 

### 4.2. QSPR Models

A limitation of most of the previously generated models is that they only consider the permeant descriptors and ignore the effect of the physicochemical properties of the vehicle or the membrane under study on the permeant diffusion process [2,5,10,35]. Such a restriction may impact upon the ability to develop representative models based on in vitro experimental data, since interactions between the vehicle and the membrane may also alter the permeation process. 

There is a good correlation between Kp and selected descriptors with r^2^ = 0.67 for the training set, which is identical to the correlation in the established Potts and Guy [2] model [2]. Comparatively, a better correlation was found between the flux and the descriptors with r^2^ of 0.72. Interrogation of Equation (2) reveals the solubility of the permeant in the oil has a positive correlation with the flux, and that those compounds that are more soluble in the oily vehicle have higher flux values. This is not surprising given the fact that the descriptors relating to the oily vehicle are the ones that dominate in the PCA plots, and this indicates that the interaction between the oil and the permeant, rather than the permeant and the membrane is one of the most important factors for determining permeation across a synthetic membrane, with this range of compounds and oily vehicles. This is not unexpected since the permeant must have sufficient solubility in order to diffuse through the membranes, and particularly so in the case of a vehicle-modified membrane. Previous studies have shown the importance of the drug solubility in the vehicles which are sorbed by the membrane [13,36].

The importance of the vehicle is also evident when examining the second descriptor in the equation, i.e., lip_acc (vehicle). This term will act to decrease the permeation coefficient of those vehicles that have greater than zero heteroatoms (O or N). That is to say, increasing the number of hydrogen bond acceptors in the vehicle decreases the flux through the membranes. In the membranes used in our modeling, silicone and polyurethane membranes are both capable of interaction and hydrogen bonding with the vehicles, which supports findings of a modification in the flux. The importance of the hydrogen bond of the drug has been reported previously in human skin and silicone [37]. The present study confirms the importance of H-bonding for the drug; however, unlike previous studies, it has been shown here that the H-bonding behavior of the vehicle is also a determinant of permeant flux across a barrier membrane. The molecular weight of the solvent also has an important effect on the uptake and mobility of the solvent inside the membrane. It has long been known that the Log K_o/w_ of the vehicle is an important descriptor for describing the solvent uptake and K_p_ [2,5]. However, it has been demonstrated that an increase in alcohol sorption by silicone membranes with a carbon number from ethanol to butanol (four carbon atoms) was observed, after which sorption decreased exponentially with increasing aliphatic chain length [13]. Additionally, it has been demonstrated that the sorption of vehicles into a membrane affects both the thermodynamic and kinetic components of co-administered compound permeation [38]. 

The solubility parameter of the vehicle is also an important predictor of the flux through the membrane. Previous studies have shown that if the solubility parameter of the vehicle is close to the solubility parameter of the skin, permeation may be enhanced [39]. Similarly, permeants with a solubility parameter close to that of the skin will have a higher permeation rate through the skin. The relationship between K_p_ of a series of alkanoic acids through porcine skin and solubility parameter was reported to take the form of a parabola with a maximum permeability near a solubility parameter of 20.46 (MPa)^1/2^ [40]. From the current study, the appearance of δH_exp drug_ parameter in Equation (2) demonstrates the importance of H-bonding contribution to the drug solubility parameter, whereby a higher value favored permeation of the drug across the model membranes. This is in agreement with findings of Cronin et al. [40] in their QSPR model for maximum steady state flux through silicone membrane. The current work has identified that matching H-bonding properties of the permeant with a vehicle component may provide a strategy to enhance molecular permeation into membranes. A molecule having a hydrogen bonding ability may have a conformational flexibility, due to its potential for intra- and inter-molecular interactions. When the membrane has become solvated by the vehicle, H-bonding compatibility between the solvent and permeant might enhance the permeation of the latter [41].

An examination of Equation (3), which describes the flux through the membrane shows six descriptors in total were important to describe the flux across synthetic membranes. Descriptors relating to the membrane, vehicle and permeant are incorporated—but the descriptors relating to the membrane are of greatest importance in explaining flux, followed by those describing the vehicle and then the permeant. The descriptors were in the order of δH_exp membrane_ >> δP_exp membrane_ > log K_o/w vehicle_ > normalized opr_brigid _vehicle_ > weight ratio > MPt_permeant_. As indicated by the equation, the most important factor (more than twice as dominant in its impact on flux as the next ranking descriptor) is δH_exp membrane_. Therefore, the greater the hydrogen bonding ability of the membrane, the bigger the effect on flux from the tested range of vehicles. This is in line with the Equation (2) results confirming that if both the vehicle and membrane are capable of the hydrogen bonding, this may increase the vehicle–membrane interaction. The resultant modification of the barrier properties of the membrane may facilitate the flux enhancement of permeants that are soluble in the vehicle. Furthermore, the equation shows that an increase in the polar component of the solubility parameter of the membrane (δP_exp membrane_) leads to an increased flux and, conversely, that the lipophilicity of the vehicle is inversely correlated with the flux. This may be due to less chances of interaction between a polar membrane and a lipophilic vehicle. 

The vehicles used in this study are all hydrophobic oils; therefore, increasing the lipophilicity of the vehicle could lead to a decrease in partition of hydrophobic drug from the oil (or oil modified membrane phase) and subsequent permeation. Equation (3) also confirms the importance of vehicle flexibility (observed in the PCA results), where there is a negative correlation between the number of rigid bonds in the vehicle and flux of the permeant across the membrane. This indicates that the less flexible the vehicle is, the lower the chances of vehicle permeation and interaction with the membrane. Moreover, the equation shows the importance of solvent uptake on the flux; the higher the vehicle uptake by a given membrane, the higher the flux of a permeant. This finding was supported by the diffusion results where a correlation was found between the amount of vehicle sorbed and the flux. The melting point of the permeant was also negatively correlated with flux. The melting point is a physicochemical property which has also been considered as a determinant of the solubility of the permeant in both the vehicle and membrane. The melting point of a permeant is highly dependent upon its intra-crystalline hydrogen bonding, confirming the importance of hydrogen bonding ability of the permeant, as well as the hydrogen-bonding interactions between vehicle and membrane. Therefore, to achieve penetration, a drug should have a low molecular weight (MW < 500 Da), moderate lipophilicity (Log *p* values 1–3), a low melting point ≤200 and few hydrogen binding points, and should be soluble in a vehicle, which has a good solvation match with the barrier membrane. Therefore, in order to induce the greatest interaction between the membrane and vehicle, the vehicle solvent should have similar partial HSP components to those of the membrane, and this can be evaluated by the HSP distance value being small.

## 5. Conclusions

These results show that the use of synthetic membranes to determine the flux of permeants from oils across epidermis should be treated with care. The diffusion results show that the vehicles containing the highest molecular branching tended to promote the permeability of the model compounds. The highest flux values for all model permeants were obtained when the compounds were suspended in IHD, and all models (PCA and QSPR) showed the importance of the steric properties of the oily vehicle on the permeation and flux of the small molecules. The diffusion of model permeants through the membranes was also correlated with the uptake of what are typically considered to be non-interacting oily vehicles into the different membranes. However, whether branched oils do interact more efficiently with these membranes does require further validation with a wider range of oily vehicles. 

The PCA analysis showed that descriptors of the oily vehicle dominated both solvent uptake and permeant K_p_. Similarly, the PCA assessment for flux using synthetic membranes identified the physicochemical properties of the vehicle as having the most influence on the rate of flux. However, the same observation was not true for the PCA assessment of flux carried out using human epidermis, where both properties of the permeant and the oily vehicle were shown to be vital to explaining the variance observed in the dataset. The QSPR models showed that it was possible to quantify the variations in permeation and flux across synthetic membranes, using simple physicochemical descriptor-based models, and once again highlighted the impact that of the properties of the oily vehicle on flux and permeation.

## Figures and Tables

**Table 1:** In vitro epidermal permeation parameters, permeability coefficients (**K_P_**) and fluxes (**J_s_**) for methyl paraben (MP), butyl paraben (BP) and caffeine (CF) from different oily vehicles and phosphate buffer pH 7.0. Data represent mean ± sd (*n* = 4 or 5).

Vehicle	J_s_ (μg cm^−2^ h^−1^)			K_P_ (10^−2^ cm h^−1^)		
	CF	MP	BP	CF	MP	BP
**Buffer**	4.22 ± 0.69	26.28 ± 0.42	17.60 ± 0.57	0.02 ± 0.002	1.09 ± 0.02	7.04 ± 0.22
**IHD**	36.60 ± 1.41	115.6 ± 10.54	86.02 ± 3.80	49.47 ± 1.90	152.61 ± 13.9	11.03 ± 0.49
**IPM**	14.56 ± 1.11	67.82 ± 2.49	56.11 ± 2.27	1.75 ± 0.13	0.19 ± 0.007	0.06 ± 0.002
**OA**	11.23 ± 0.53	56.27 ± 1.06	54.05 ± 1.49	0.21 ± 0.010	0.86 ± 0.02	0.09 ± 0.002
**HD**	9.43 ± 0.53	47.78 ± 2.51	44.61 ± 4.61	12.74± 0.71	60.47 ± 3.17	5.95 ± 0.61
**LP**	9.66 ± 0.46	43.24 ± 2.94	26.98 ± 1.20	12.54 ± 0.60	61.77 ± 4.19	3.70 ± 0.16

**Table 2:** Physicochemical descriptors, categorized according to whether they are related to the vehicle, permeant or membrane, that were identified by Principal Components Analysis (PCA) as important for explaining the variation in experimental observation for the solvent uptake, flux through membranes, and permeability coefficient through synthetic membranes and flux through epidermis.

Experiment	Physicochemical Descriptors Relating to the Vehicle	Physicochemical Descriptors Relating to the Permeant	Physicochemical Descriptors Relating to the Membrane
Solvent uptake	δ_D_(vehicle); Log K_o/w_(vehicle); molecular connectivity index (vehicle); opr_brigid (vehicle); chi0_C (vehicle).	-	HSP distance between the oils and membranes
Flux through synthetic membrane	LogK_o/w_(vehicle); lip_acc (vehicle); lip_don (vehicle); chi1_C (vehicle).	solubility of the permeants in the oils; δ_P_ (permeant).	weight ratio of treated: untreated membranes; Hansen Solubility Parameter (HSP) distance between the oils and membranes.
Permeability through synthetic membrane	Mpoint (vehicle); Log K_o/w_(vehicle); molecular connectivity index (vehicle); opr_brigid (vehicle); chi0 (vehicle).	solubility of the permeants in the oils; Mpoint (permeant).	weight ratio of treated: untreated membranes; HSP distance between the oils and membranes.
Flux through epidermis	density (vehicle); opr_brigid (vehicle); Kier flex (vehicle).	solubility of the permeants in the oils; opr_nring (permeant); chi0 (permeant); chi0_C (permeant).	-

## Supplementary Materials

The following are available online at https://www.mdpi.com/1999-4923/12/10/958/s1, Appendix I: Definitions of molecular descriptors used in PCA analysis.

## References

[1] Flynn G.L., Gerrity Timothy H.C. (1990). Physicochemical determinants of skin absorption. Principles of Route-To-Route Extrapolation for Risk Assessment.

[2] Potts R.O., Guy R.H. (1992). Predicting skin permeability. Pharm. Res..

[3] Wilschut A., ten Berge W.F., Robinson P.J., McKone T.E. (1995). Estimating skin permeation. The validation of five mathematical skin permeation models. Chemosphere.

[4] Pugh W. (1996). Epidermal permeability—Penetrant structure relationships: 3. The effect of hydrogen bonding interactions and molecular size on diffusion across the stratum corneum. Int. J. Pharm..

[5] Moss G.P., Cronin M.T.D. (2002). Quantitative structure–permeability relationships for percutaneous absorption: Re-analysis of steroid data. Int. J. Pharm..

[6] Mitragotri S. (2003). Modeling skin permeability to hydrophilic and hydrophobic solutes based on four permeation pathways. J. Control. Release.

[7] Mitragotri S., Anissimov Y.G., Bunge A.L., Frasch H.F., Guy R.H., Hadgraft J., Kasting G.B., Lane M.E., Roberts M.S. (2011). Mathematical models of skin permeability: An overview. Int. J. Pharm..

[8] Chen L., Han L., Lian G. (2013). Recent advances in predicting skin permeability of hydrophilic solutes. Adv. Drug Deliv. Rev..

[9] Hathout R.M. (2014). Using principal component analysis in studying the transdermal delivery of a lipophilic drug from soft nano-colloidal carriers to develop a quantitative composition effect permeability relationship. Pharm. Dev. Technol..

[10] Potts R.O., Guy R.H. (1995). A predictive algorithm for skin permeability: The effects of molecular size and hydrogen bond activity. Pharm. Res..

[11] Sloan K.B., Siver K.G., Koch S.A.M. (1986). The Effect of Vehicle on the Diffusion of Salicylic Acid Through Hairless Mouse Skin. J. Pharm. Sci..

[12] Golden G.M., McKie J.E., Potts R.O. (1987). Role of Stratum Corneum Lipid Fluidity in Transdermal Drug Flux. J. Pharm. Sci..

[13] McAuley W.J., Lad M.D., Mader K.T., Santos P., Tetteh J., Kazarian S.G., Hadgraft J., Lane M.E. (2010). ATR-FTIR spectroscopy and spectroscopic imaging of solvent and permeant diffusion across model membranes. Eur. J. Pharm. Biopharm..

[14] Haware R.V., Tho I., Bauer-Brandl A. (2009). Application of multivariate methods to compression behavior evaluation of directly compressible materials. Eur. J. Pharm. Biopharm..

[15] Shi S., Dodds Ashley E.S., Alexander B.D., Hickey A.J. (2009). Initial Characterization of Micafungin Pulmonary Delivery via Two Different Nebulizers and Multivariate Data Analysis of Aerosol Mass Distribution Profiles. AAPS PharmSciTech.

[16] Yi Z.-B., Yan Y., Liang Y.-Z., Zeng B. (2007). Evaluation of the antimicrobial mode of berberine by LC/ESI-MS combined with principal component analysis. J. Pharm. Biomed. Anal..

[17] Basak S.C., Mills D., Mumtaz M.M. (2007). A quantitative structure–activity relationship (QSAR) study of dermal absorption using theoretical molecular descriptors. SAR QSAR Environ. Res..

[18] CHEN L., LIAN G., HAN L. (2007). Prediction of human skin permeability using artificial neural network (ANN) modeling. Acta Pharmacol. Sin..

[19] Patel J. (2013). Science of the Science, Drug Discovery and Artificial Neural Networks. Curr. Drug Discov. Technol..

[20] Baba H., Takahara J.I., Mamitsuka H. (2015). In silico predictions of human skin permeability using nonlinear quantitative structure-property relationship models. Pharm. Res..

[21] Keurentjes A.J., Maibach H.I. (2019). Percutaneous penetration of drugs applied in transdermal delivery systems: An in vivo based approach for evaluating computer generated penetration models. Regul. Toxicol. Pharmacol..

[22] Lefnaoui S., Rebouh S., Bouhedda M., Yahoum M.M. (2020). Artificial neural network for modeling formulation and drug permeation of topical patches containing diclofenac sodium. Drug Deliv. Transl. Res..

[23] Tsakovska I., Pajeva I., Al Sharif M., Alov P., Fioravanzo E., Kovarich S., Worth A.P., Richarz A.-N., Yang C., Mostrag-Szlichtyng A. (2017). Quantitative structure-skin permeability relationships. Toxicology.

[24] Najib O.N., Martin G.P., Kirton S.B., Sallam A.-S., Murnane D. (2016). Establishing the importance of oil-membrane interactions on the transmembrane diffusion of physicochemically diverse compounds. Int. J. Pharm..

[25] Jackson J.E. (1991). A Use’s Guide to Principal Components.

[26] Tracy N.D., Young J.C., Mason R.L. (1992). Multivariate Control Charts for Individual Observations. J. Qual. Technol..

[27] Postnov V.V., Gafarova N.A., Serikov Z.S., Nauruzov M.K., Malenko E.V. (1972). Composition of liquid paraffinic hydrocarbons from Mangyshlak crude. Chem. Technol. Fuels Oils.

[28] Hansen C.M. (1967). The Three Dimensional Solubility Parameter and Solvent Diffusion Coefficient, Their Importance in Surface Coating Formulation.

[29] Cronin M.T.D., Dearden J.C., Gupta R., Moss G.P. (1998). An investigation of the mechanism of flux across polydimethylsiloxane membranes by use of quantitative structure-permeability relationships. J. Pharm. Pharmacol..

[30] Feldstein M.M., Raigorodskii I.M., Iordanskii A.L., Hadgraft J. (1998). Modeling of percutaneous drug transport in vitro using skin-imitating Carbosil membrane. J. Control. Release.

[31] Russeau W., Mitchell J., Tetteh J., Lane M.E., Hadgraft J. (2009). Investigation of the permeation of model formulations and a commercial ibuprofen formulation in Carbosil® and human skin using ATR-FTIR and multivariate spectral analysis. Int. J. Pharm..

[32] Zadeh B.S.M., Moghimi H., Santos P., Hadgraft J., Lane M.E. (2008). A comparative study of the in vitro permeation characteristic of sulphadiazine across synthetic membranes and eschar tissue. Int. Wound J..

[33] Stamatas G.N., de Sterke J., Hauser M., von Stetten O., van der Pol A. (2008). Lipid uptake and skin occlusion following topical application of oils on adult and infant skin. J. Dermatol. Sci..

[34] Zhang Q., Li P., Roberts M.S. (2011). Maximum transepidermal flux for similar size phenolic compounds is enhanced by solvent uptake into the skin. J. Control. Release.

[35] Lam L.T., Sun Y., Davey N., Adams R., Prapopoulou M., Brown M.B., Moss G.P. (2010). The application of feature selection to the development of Gaussian process models for percutaneous absorption. J. Pharm. Pharmacol..

[36] Dias M., Raghavan S.L., Hadgraft J. (2001). ATR-FTIR spectroscopic investigations on the effect of solvents on the permeation of benzoic acid and salicylic acid through silicone membranes. Int. J. Pharm..

[37] Cronin M.T.D., Dearden J.C., Moss G.P., Murray-Dickson G. (1999). Investigation of the mechanism of flux across human skin in vitro by quantitative structure–permeability relationships. Eur. J. Pharm. Sci..

[38] Oliveira G., Beezer A.E., Hadgraft J., Lane M.E. (2010). Alcohol enhanced permeation in model membranes. Part I. Thermodynamic and kinetic analyses of membrane permeation. Int. J. Pharm..

[39] Dias M., Hadgraft J., Lane M. (2007). Influence of membrane–solvent–solute interactions on solute permeation in skin. Int. J. Pharm..

[40] Liron Z., Cohen S. (1984). Percutaneous Absorption of Alkanoic Acids 11: Application of Regular Solution Theory. J. Pharm. Sci..

[41] McAuley W.J., Mader K.T., Tetteh J., Lane M.E., Hadgraft J. (2009). Simultaneous monitoring of drug and solvent diffusion across a model membrane using ATR-FTIR spectroscopy. Eur. J. Pharm. Sci..

