# Supplementary material for: Multivariate Analytical Approaches to Identify Key Molecular Properties of Vehicles, Permeants and Membranes That Affect Permeation through Membranes
Source: Pharmaceutics. 2020 Oct 11;12(10):958. doi: 10.3390/pharmaceutics12100958 (PMC7599860; doi:10.3390/pharmaceutics12100958)
Supplement: Supplementary file 1 [file pharmaceutics-12-00958-s001.pdf]

pharmaceutics

Supplementary Materials: Multivariate Analytical Approaches to Identify Key Molecular Properties of Vehicles, Permeants and Membranes That Affect Permeation through Membranes

Omaima N. Najib, Stewart B. Kirton, Gary P. Martin, Michelle J. Botha, Al-Sayed Sallam and Darragh Murnane

Appendix I: Definitions of molecular descriptors used in PCA analysis

The molecular descriptors used in PCA analysis were determined using Molecular Operating Environment 2012, produced by Chemical Computing Group Inc., Montreal, Canada. and by HSPiP software version 4.0.04

Code	Description
Solvent uptake	Amount of oil sorbed by membrane
solubility	Solubility of the permeants in the oil
density	Molecular mass density: Weight divided by vdw_vol (amu/Å^3)
Weight	Molecular weight (including implicit hydrogens) in atomic mass units with atomic weights taken from [1]
Mpoint	Melting point
δ_D	The dispersive component of HSP
δ_P	The polar component of HSP
δ_H	The hydrogen bond component of HSP
δ_T	Hildebrand solubility parameter
LogP (o/w)	Log of the octanol/water partition coefficient (including implicit hydrogens). This property is calculated from a linear atom type model with r^2 = 0.931, RMSE=0.393 on 1,827 molecules
Log $K_{o/w}$	Log of the octanol/water partition coefficient (including implicit hydrogens). This property is an atomic contribution model that calculates LogP from the given structure; i.e., the correct protonation state (washed structures). Results may vary from the LogP(o/w) descriptor. The training set for SlogP was ~7000 structures
ovality	A measure of how the shape of a molecule approaches an oval shape
Mwt	Molecular weight
Mvol	Molecular volume
TPSA	Polar surface area (Å2) calculated using group contributions to approximate the polar surface area from connection table

	information only. The parameterization is that of Ertl et al. [2]
vdw_vol	van der Waals volume (Å3) calculated using a connection table approximation
vdw_area	Area of van der Waals surface (Å2) calculated using a connection table approximation
a_nH	Number of hydrogen atoms (including implicit hydrogens). This is calculated as the sum of hi over all non-trivial atoms i plus the number of non-trivial hydrogen atoms
a_nC	Number of carbon atoms: #{Zi | Zi = 6}
a_nO	Number of oxygen atoms: #{Zi | Zi = 8}
b_1rotN	Number of rotatable single bonds. Conjugated single bonds are not included (e.g., ester and peptide bonds).
b_1rotR	Fraction of rotatable single bonds: b_1rotN divided by b_heavy
b_ar	Number of aromatic bonds.
b_count	Number of bonds (including implicit hydrogens). This is calculates the sum of (di/2 + hi) over all non-trivial atoms i
b_double	Number of double bonds. Aromatic bonds are not considered to be double bonds
b_rotN	Number of rotatable bonds. A bond is rotatable if it has order 1, is not in a ring, and has at least two heavy neighbors
b_rotR	Fraction of rotatable bonds: b_rotN divided by b_heavy
b_single	Number of single bonds (including implicit hydrogens). Aromatic bonds are not considered to be single bonds
lip_acc	The number of O and N atoms
lip_don	The number of OH and NH atoms
opr_brigid	The number of rigid bonds [3]
opr_nring	The number of ring bonds [3]
opr_nrot	The number of rotatable bonds from [3]
chi0	Atomic connectivity index (order 0) [4,5]. This is calculated as the sum of 1/sqrt(di) over all heavy atoms i with di > 0
chi0_C	Carbon connectivity index (order 0). This is calculated as the sum of 1/sqrt(di) over all carbon atoms i with di > 0
chi1	Atomic connectivity index (order 1) [4,5]. This is calculated as the sum of 1/sqrt(didj) over all bonds between heavy atoms i and j where i < j
chi1_C	Carbon connectivity index (order 1). This is calculated as the sum

	of 1/sqrt(didj) over all bonds between carbon atoms i and j where i < j
chi0v	Atomic valence connectivity index (order 0) [4,5]. This is calculated as the sum of 1/sqrt(vi) over all heavy atoms i with vi > 0
chi0v_C	Carbon valence connectivity index (order 0). This is calculated as the sum of 1/sqrt(vi) over all carbon atoms i with vi > 0
Kier1	First kappa shape index: (n-1)2 / m^2 [5]
Kier2	Second kappa shape index: (n-1)2 / m^2 [5]
Kier3	Third kappa shape index: (n-1) (n-3)2 / p32 for odd n, and (n-3) (n-2)2 / p32 for even n (Hall, 1991)
KierFlex	Kier molecular flexibility index: (KierA1) (KierA2) / n [5]
balabanJ	Balaban's connectivity topological index [6]
Q_VSA_HYD PEOE_VSA_HYD	Total hydrophobic van der Waals surface area. This is the sum of the vi such that |qi| is less than or equal to 0.2. The vi are calculated using a connection table approximation.
Q_VSA_FPOL PEOE_VSA_FPOL	Fractional polar van der Waals surface area. This is the sum of the vi such that |qi| is greater than 0.2 divided by the total surface area. The vi are calculated using a connection table approximation
Q_VSA_FHYD PEOE_VSA_FHYD	Fractional hydrophobic van der Waals surface area. This is the sum of the vi such that |qi| is less than or equal to 0.2 divided by the total surface area. The vi are calculated using a connection table approximation
Q_VSA_POL PEOE_VSA_POL	Total polar van der Waals surface area. This is the sum of the vi such that |qi| is greater than 0.2. The vi are calculated using a connection table approximation

References

1. *CRC handbook of chemistry and physics*; CRC Press, ©1994;
2. Ertl, P.; Rohde, B.; Selzer, P. Fast Calculation of Molecular Polar Surface Area as a Sum of Fragment-Based Contributions and Its Application to the Prediction of Drug Transport Properties. *J. Med. Chem.* **2000**, *43*, 3714–3717, doi:10.1021/jm000942e.
3. Oprea, T.I. Property distribution of drug-related chemical databases. *J. Comput. Aided. Mol. Des.* **2000**, doi:10.1023/A:1008130001697.
4. Kier, L.B.; Hall, L.H. The nature of structure-activity relationships and their relation to molecular connectivity. *Eur. J. Med. Chem.* **1977**.
5. Hall, L.H.; Kier, L.. The Molecular Connectivity Chi Indices and Kappa Shape Indices in Structure-Property Modeling. In *Reviews in Computational Chemistry*; Boyd, enny B.L.D.B., Ed.; 1991.
6. Balaban, A.T. Highly discriminating distance-based topological index. *Chem. Phys. Lett.* **1982**, *89*, 399–404, doi:10.1016/0009-2614(82)80009-2.
